# Image set for deep learning: field images of maize annotated with disease symptoms

**DOI:** 10.1186/s13104-018-3548-6

**Published:** 2018-07-03

**Authors:** Tyr Wiesner-Hanks, Ethan L. Stewart, Nicholas Kaczmar, Chad DeChant, Harvey Wu, Rebecca J. Nelson, Hod Lipson, Michael A. Gore

**Affiliations:** 1 Plant Breeding and Genetics Section, School of Integrative Plant Science, Cornell University, Ithaca, NY 14853 USA; 2 Department of Computer Science, Columbia University, New York, NY 10027 USA; 3 Plant Pathology and Plant–Microbe Biology Section, School of Integrative Plant Science, Cornell University, Ithaca, NY 14853 USA; 4 Department of Mechanical Engineering and Institute of Data Science, Columbia University, New York, NY 10027 USA

**Keywords:** Plant disease, Maize, Corn, Disease, Phytopathology, Machine learning, Convolutional neural network, Images, Deep learning

## Abstract

**Objectives:**

Automated detection and quantification of plant diseases would enable more rapid gains in plant breeding and faster scouting of farmers’ fields. However, it is difficult for a simple algorithm to distinguish between the target disease and other sources of dead plant tissue in a typical field, especially given the many variations in lighting and orientation. Training a machine learning algorithm to accurately detect a given disease from images taken in the field requires a massive amount of human-generated training data.

**Data description:**

This data set contains images of maize (*Zea mays* L.) leaves taken in three ways: by a hand-held camera, with a camera mounted on a boom, and with a camera mounted on a small unmanned aircraft system (sUAS, commonly known as a drone). Lesions of northern leaf blight (NLB), a common foliar disease of maize, were annotated in each image by one of two human experts. The three data sets together contain 18,222 images annotated with 105,705 NLB lesions, making this the largest publicly available image set annotated for a single plant disease.

## Objective

Globally, plant diseases are an enormous burden to farmers. Northern leaf blight (NLB), a foliar disease of maize, has become increasingly severe in the US [1]. Screening a large area for early symptoms is time-consuming, and there is high intra- and inter-rater variation in NLB severity estimates [2].

Automated, field-based detection of plant disease symptoms would be valuable for plant breeders and growers. However, this is complicated by the “noisy” nature of field imagery. There may be many sources of dead tissue, along with obscured symptoms. This requires a computer vision approach that is specific to the target disease and insensitive to such variations.

Convolutional neural networks (CNNs) are a class of machine learning models that can be trained to accurately detect objects in images, making them the current standard for object recognition [3]. CNNs must be trained on a large number of classified or annotated images, but unlike recognizable everyday objects, plant disease symptoms require expertise and experience to identify.

Very few large, expert-curated image sets of plant disease exist [4]. PlantVillage contains over 50,000 images of numerous crops and diseases [5]. However, these were taken with detached leaves on a plain background, and CNNs trained on these did not perform well on field images [6]. Other image sets are much smaller [7], or not curated by experts [8].

We collected image data from several platforms and angles to help develop a system for real-time monitoring and phenotyping of NLB in maize fields using drones equipped with CNNs. The resulting data set exceeds 18,000 maize plant images annotated with more than 100,000 NLB lesions, which is the largest collection of images for any one plant disease. These annotated images are expected to be valuable for furthering the development of novel computer vision and deep learning approaches in agriculture.

## Data description

The data consists of three image sets and their accompanying annotations. All images were taken in field trials of maize that had been inoculated with *Setosphaeria turcica*, the causal agent of NLB. All trials were planted at Cornell University’s Musgrave Research Farm in Aurora, NY (https://cuaes.cals.cornell.edu/farms/musgrave-research-farm/). The trials consisted of maize hybrids from The Genomes to Fields Initiative (https://www.genomes2fields.org/resources/), arranged in two-row plots with a length of 5.64 m and inter-row spacing of 0.76 m. There was a 0.76 m alley at the end of each plot. The trials were rainfed and managed with conventional maize cultivation practices. Plants were inoculated at the V5–V6 stage with both a liquid suspension of *S. turcica* (isolate NY001) spores and sorghum grains colonized by the fungus as previously described [9]. The first image set, the “handheld set,” was taken by hand in summer 2015. This image set was described and analyzed previously [9], but is included here to make all images available in a single repository. The second, the “boom set,” was taken by mounting the camera on a 5 m boom in summer 2015. This boom held the remotely triggered camera above the canopy with nadir view. The third, the “drone set,” was taken by mounting the camera on a DJI Matrice 600 sUAS in summer 2017. The drone was flown at an altitude of 6 m and a velocity of 1 m/s, and images were captured with nadir view every 2 s.

For the handheld and boom sets, images were checked manually to ensure the image was in focus and otherwise adequate. For the drone set, images with a low total length of edges (as reported by canny edge detection) were filtered out, in order to remove blurry images. Images were then discarded during annotation if they were out of focus or otherwise unacceptable.

In each image, lesions were annotated by one of two human experts, as denoted in the annotation files. Annotators drew a line down the main axis of each lesion visible in the image, stretching down the entire length of the lesion. If a lesion appeared bent or curved from the camera’s perspective, two or more intersecting annotation lines were drawn to form an angle or arc as needed. In the handheld set, this was done with the markup tools in Bisque [9]. In the boom and drone sets, these steps were done using a custom ImageJ macro (Table 1, lesionCount_v2.1_dataNote.txt). Endpoint coordinates of each annotation line are recorded in the three.csv data files, each corresponding to a single data set (Table 1). Images with 0 values for all four endpoint coordinates had no visible lesions.

**Table 1:** Overview of data files/data sets

Label	Name of data file/set	File type (extension)	Data repository and identifier
Images	images_handheld	.tar.gz (folder with.jpg files)	https://osf.io/arwmy/
	images_boom	.tar.gz (folder with.jpg files)	https://osf.io/er3zb/
	images_drone	.tar.gz (folder with.jpg files)	https://osf.io/vfawp/
Annotations	annotations_handheld	.csv	https://osf.io/7ue84/
	annotations_boom	.csv	https://osf.io/u6mfb/
	annotations_drone	.csv	https://osf.io/25agh/
ImageJ macro	lesionCount_v2.1_dataNote	.txt	https://osf.io/av7dj/

The number of images and annotation lines are as follows:

- Handheld: 1787 images, 7669 annotations.
- Boom: 8766 images, 55,919 annotations.
- Drone: 7669 images, 42,117 annotations.

Some boom images are 1/4 slices of larger images, as a wider field of view made it difficult to annotate the entire image at once. These are denoted with suffixes, e.g., ‘img01_00.jpg’, ‘img01_01.jpg.’

## Limitations

- Lesion axis annotations do not indicate width or margins.
- There is no way to indicate confidence of annotations. Some lesions are easily visible, while others are partially occluded, out of the main focal plane, in heavy shade, or washed out by bright sunlight.
- Even experts may have a hard time distinguishing between NLB and similar-looking diseases, such as Stewart’s wilt or anthracnose leaf blight, from a distance. While no similar-looking diseases were noted as we phenotyped fields on foot, this does not preclude the possibility of such false positives.
- All photographs were taken in a single field in central New York State. This limits the generalizability of the data, as symptoms of the same disease in other regions may present or develop differently.

## Abbreviations

CNN: convolutional neural network
NLB: northern leaf blight
sUAS: small unmanned aircraft system
